# Diagnostic and Prognostic Impact of Circulating YKL-40, IL-6, and CA 19.9 in Patients with Pancreatic Cancer

**DOI:** 10.1371/journal.pone.0067059

**Published:** 2013-06-26

**Authors:** Nicolai A. Schultz, Ib J. Christensen, Jens Werner, Nathalia Giese, Benny V. Jensen, Ole Larsen, Jon K. Bjerregaard, Per Pfeiffer, Dan Calatayud, Svend E. Nielsen, Mette K. Yilmaz, Niels H. Holländer, Morten Wøjdemann, Stig E. Bojesen, Kaspar R. Nielsen, Julia S. Johansen

**Affiliations:** 1 Department of Surgical Gastroenterology and Transplantation, Rigshospitalet, University of Copenhagen, Copenhagen, Denmark; 2 The Finsen Laboratory, Rigshospitalet, and Biotech Research and Innovation Centre (BRIC), University of Copenhagen, Copenhagen, Denmark; 3 Department of General, Visceral, and Transplant Surgery, University of Heidelberg, Heidelberg, Germany; 4 Department of Oncology Copenhagen University Hospital at Herlev, Denmark; 5 Department of Oncology Odense University Hospital, Odense, Denmark; 6 Hillerød Hospital, Hillerød, Denmark; 7 Department of Oncology Aalborg University Hospital, Aalborg, Denmark; 8 Department of Oncology Næstved Hospital, Næstved, Denmark; 9 Department of Surgical Gastroenterology, Copenhagen University Hospital at Herlev, Herlev, Denmark; 10 Department of Clinical Biochemistry, Copenhagen University Hospital at Herlev, Herlev, Denmark; 11 Department of Medicine, Copenhagen University Hospital at Herlev, Herlev, Denmark; 12 Department of Clinical Immunology, Aalborg University Hospital, Aalborg, Denmark; MOE Key Laboratory of Environment and Health, School of Public Health, Tongji Medical College, Huazhong University of Science and Technology, China

## Abstract

**Purpose:**

We tested the hypothesis that high plasma YKL-40 and IL-6 associate with pancreatic cancer and short overall survival.

**Patients and Methods:**

In all, 559 patients with pancreatic cancer from prospective biomarker studies from Denmark (n = 448) and Germany (n = 111) were studied. Plasma YKL-40 and IL-6 were determined by ELISAs and serum CA 19.9 by chemiluminescent immunometric assay.

**Results:**

Odds ratios (ORs) for prediction of pancreatic cancer were significant for all biomarkers, with CA 19.9 having the highest AUC (CA 19.9: OR = 2.28, 95% CI 1.97 to 2.68, p<0.0001, AUC = 0.94; YKL-40: OR = 4.50, 3.99 to 5.08, p<0.0001, AUC = 0.87; IL-6: OR = 3.68, 3.08 to 4.44, p<0.0001, AUC = 0.87). Multivariate Cox analysis (YKL-40, IL-6, CA 19.9, age, stage, gender) in patients operated on showed that high preoperative IL-6 and CA 19.9 (dichotomized according to normal values) were independently associated with short overall survival (CA 19.9: HR = 2.51, 1.22–5.15, p = 0.013; IL-6: HR = 2.03, 1.11 to 3.70, p = 0.021). Multivariate Cox analysis of non-operable patients (Stage IIB-IV) showed that high pre-treatment levels of each biomarker were independently associated with short overall survival (YKL-40: HR = 1.30, 1.03 to 1.64, p = 0.029; IL-6: HR = 1.71, 1.33 to 2.20, p<0.0001; CA 19.9: HR = 1.54, 1.06 to 2.24, p = 0.022). Patients with preoperative elevation of both IL-6 and CA 19.9 had shorter overall survival (p<0.005) compared to patients with normal levels of both biomarkers (45% vs. 92% alive after 12 months).

**Conclusions:**

Plasma YKL-40 and IL-6 had less diagnostic impact than CA 19.9. Combination of pretreatment YKL-40, IL-6, and CA 19.9 may have clinical value to identify pancreatic cancer patients with the poorest prognosis.

## Introduction

Surgery is the only potential curative therapy for patients with pancreatic cancer (PC). Most patients have locally advanced or metastatic PC at time of diagnosis and surgery is possible in less than 20% [1]. New biomarkers for early diagnosis and guidance of treatment for patients with PC are urgently needed [2]. Not all patients with PC have elevated serum CA 19.9, but this biomarker has a prognostic value and is used mainly to guide treatment and follow-up of patients with PC [3], [4].

Plasma concentrations of YKL-40 and IL-6 are emerging as new biomarkers in patients with cancer (5,6). YKL-40 (chitinase 3-like 1, CHI3L1) is a highly conserved glycoprotein produced by cancer cells (including PC), macrophages, neutrophils, and by fetal and embryonic stem cells (5,7–9). IL-6 and hypoxia stimulate YKL-40 production (10,11). YKL-40 regulates vascular endothelial growth factor (12,13), activates Akt signaling [20], protects against apoptosis [21], and plays a role in inflammation [5], [14], [15], bacterial clearance during infections [16], angiogenesis [12], [13], [17]–[19], cell proliferation and differentiation [8], [9], and remodeling of the extracellular matrix [5]. Plasma YKL-40 is elevated in some patients with PC, and in a proximity ligation assay study, the combination of plasma YKL-40, osteopontin, and CA 19-9 improved the diagnostic accuracy compared to CA 19-9 alone [22]. Furthermore, high plasma YKL-40 in subjects from the general population is associated with increased risk of and death from gastrointestinal cancer, including PC [23].

Interleukin-6 (IL-6) is produced by cancer cells (including PC), macrophages, lymphocytes, and endothelial cells [6], [24]–[26]. IL-6 plays a role in inflammation, acute-phase response, and development of cachexia, acts as a paracrine and autocrine growth factor for cancer cells, and inhibits radio- and chemotherapy-induced apoptosis of PC cells [6], [25], [27], [28]. High plasma IL-6 is associated with poor prognosis in patients with PC and is an independent prognostic biomarker [29].

A pilot study has shown that plasma concentrations of YKL-40 and IL-6 are elevated in patients with upper gastrointestinal cancers including patients with PC. Furthermore, changes in plasma YKL-40 and IL-6 during treatment were related to outcome [30]. In the present study, we tested the hypotheses: 1) elevated plasma concentrations of YKL-40 and IL-6 in combination with serum CA 19.9 can be used to diagnose patients with PC; and 2) elevated pre-treatment plasma YKL-40 and IL-6 predict poor prognosis. To do this, we studied 559 patients with PC from two large prospective biomarker studies in Denmark and Germany.

## Patients and Methods

### Patient Characteristics

#### BIOPAC study

From July 1, 2008 through June 30, 2012, pretreatment blood samples were collected from 448 patients diagnosed with PC recruited consecutively from six hospitals in Denmark (Herlev University Hospital n = 224, Odense University Hospital n = 94, Rigshospitalet n = 57, Hillerød Hospital n = 29, Aalborg Hospital n = 22, and Næstved Hospital n = 22). The patients were included in the Danish multicenter BIOPAC Study “BIOmarkers in patients with PAncreatic Cancer (BIOPAC) – can they provide new information of the disease and improve diagnosis and prognosis of the patients?”. Clinical eligibility criteria for inclusion were age over 18 years and histological or cytological confirmed pancreatic adenocarcinomas. Forty-two patients with local PC underwent a pancreaticoduodenectomy, a distal pancreatectomy, or a total pancreatectomy, and 37 of these patients received adjuvant gemcitabine after operation. Three-hundred-ninety patients with locally advanced or metastatic PC received first line palliative gemcitabine (n = 361), gemcitabine in combination with capecitabine (n = 17), gemcitabine in combination with erlotinib (n = 1), 5-FU in combination with irinotecan and oxaliplatin (n = 6), or chemo-radio therapy (n = 5) until disease progression. Sixteen patients (all non-operable) received no treatment. Table 1 shows the clinical characteristics of the patients with PC. Patients were followed until death or October 6, 2012. CT scans were performed every 3^rd^ month or on suspicion of disease progression. All patients provided written informed consent. The “BIOPAC Study” was approved by the Regional Ethics Committee (VEK ref. KA-20060094) and the Danish Data Protection Agency.

**Table 1:** Clinical characteristics of 559 patients with PC in whom at least one biomarker was available.

Variable	BIOPAC Cohort (n = 448)	Heidelberg Cohort (n = 111)
**Age**	67 (37–89)	62 (31–84)
**Sex, male/female**	256/192	63/48
**Stage, IA/IB/IIA/IIB/III/IV/unknown**	7/6/23/47/90/267/8	0/0/20/79/3/7/2
**Number of operated patients and non-operable patients**	42/406	110/1

#### Heidelberg study

From August 2003 through November 2009, pretreatment blood samples were collected from 111 patients diagnosed with PC and recruited consecutively at Department of General, Visceral, and Transplant Surgery, University of Heidelberg, Germany. Clinical eligibility criteria for inclusion were age over 18 years, histologically confirmed PC, undergone surgery with radical intention for PC, and adequate organ function. Only one of the patients was found non-resectable during surgery. After operation, 107 patients received adjuvant gemcitabine. Patients were followed until death or October 7, 2011. All patients provided written informed consent, and the study (including biomarker analysis) was approved by the regional ethics committee.

### Biomarker Analysis

Standard operating procedures were used for blood sampling. Blood for serum and EDTA plasma were centrifuged within ½-2 hours after blood sampling and then stored at −80°C until analysis. Plasma concentrations of YKL-40 and IL-6 were determined in duplicate by commercial enzyme-linked immunosorbent assays (ELISA) (YKL-40: Quidel, Santa Clara, CA, USA; IL-6: Catalogue number HS600, R&D Systems, Abingdon, Oxon, UK) according to the manufacturers’ instructions. YKL-40 ELISA characteristics: Detection limit 20 µg/l, and intra- and inter-assay coefficients of variation (CVs) were <5% and <6% [30]. IL-6 ELISA characteristics: detection limit 0.01 ng/l, and intra- and inter-assay CVs were ≤ 8% and ≤ 11% [31]. Serum CA 19.9 was analyzed using the Immulite 2000 GI-MA (Siemens, Catalogue Number L2KG12) assay, a solid-phase, two-site sequential chemiluminescent immunometric assay.

### YKL-40, IL-6, and CA 19.9 in Healthy Subjects and Patients with Chronic Pancreatitis

#### Healthy subjects

The reference interval for plasma YKL-40 was determined in 3,130 healthy subjects (1293 men, 1837 women) aged 21–84 years from the Danish general population, the Copenhagen City Heart Study. They had no known disease at time of blood sampling in 1991–1994 and remained healthy and alive during the 16-year follow-up period [31]. The median plasma YKL-40 in these 3,130 healthy subjects was 40 µg/l (min-max: 20–1098 µg/l; 5–95% percentile range, 20 to 116 µg/l). The reference interval for plasma IL-6 was determined in 318 healthy blood donors (196 men, 122 women) aged 18–64 years. These subjects were all healthy, were not on medication, and had no signs or clinical symptoms of disease. The median plasma IL-6 in these 306 healthy subjects was 1.3 ng/l (min-max: 0.33–26 ng/l; 5–95% percentile range, 0.63 to 4.50 ng/l) [32]. The upper normal limit for serum CA 19.9 was 37 KU/l according to the manufacturer. The reference intervals for serum CA 19.9 for the ROC curves were determined in 142 healthy blood donors (72 men, 70 women) aged 41–66 years (median 57 years). These subjects were healthy, were not on medication, and had no signs or clinical symptoms of disease. The median serum CA 19.9 in these 142 healthy subjects was 4.25 KU/l (min–max: 2.50–77 KU/l; 5–95% percentile range, 2.50 to 28 KU/l).

#### Patients with chronic pancreatitis

The reference intervals for plasma YKL-40, IL-6 and serum CA 19.9 were determined in 80 patients with chronic pancreatitis (58 men, 22 women) aged 32–85 years. The median plasma YKL-40 was 102 µg/l (min-max: 20–1661 µg/l; 5–95% percentile range, 22 to 1047 µg/l). The median serum IL-6 was 3.5 ng/l (min–max: 0.51–64.7 ng/l; 5–95% percentile range, 0.81 to 12.75 ng/l), and the median serum CA 19.9 was 15 KU/l (min–max: 2.5–1134 KU/l; 5–95% percentile range, 2.5 to 287 KU/l).

### Statistical Analysis

The results of this project are reported in accordance with the REMARK guidelines [33]. Descriptive statistics for continuous variables are presented by their median levels and range. Analyses comparing patients to normal reference levels were done using logistic regression analysis. Results are presented by the receiver operating characteristics curve (ROC) and discrimination assessed by the area under the ROC (AUC). Similar analyses were done on subsets of patients. For correlations we used Spearman’s rank correlations.

Duration of survival was updated October 6, 2012 (BIOPAC Study) and October 7, 2011 (Heidelberg Study). Cases in which patients were alive on this date were censored. The primary endpoint was overall survival (OS). Survival probabilities for OS were estimated by the Kaplan-Meier method, and tests for differences between strata were done using the log-rank statistic. Graphical presentation using Kaplan-Meier estimates of OS was shown grouping patients by high vs. normal plasma YKL-40, IL-6, and serum CA 19.9 level. The Cox proportional hazards model was used for analysis of time to death, adjusting the model for stage, age, and gender. The levels of the biomarkers were analyzed either by the dichotomized value using the 95^th^ percentile of reference data as the cut-point or by the actual value of the biomarker on the log scale (log base 2). Univariate and multivariate analyses were performed. Tests for potential interactions were done where applicable. Model assessment was done using graphical methods, Schoenfeld and Martingale residuals. P-values less than 5% were considered significant. All calculations were performed using SAS (version 9.1, SAS Institute, Cary, NC, USA).

## Results

### YKL-40, IL-6 and CA 19.9 and Risk of PC

Table 1 gives the clinical characteristics of the 559 patients with PC. Pre-treatment plasma YKL-40, IL-6, and serum CA 19.9 were elevated in 36%, 54%, and 82% of all patients with PC, respectively. Plasma YKL-40, IL-6 and serum CA 19.9 increased with increasing stage (Supplementary Table 1 and Supplementary Figure 1). Elevated serum CA 19.9 was found in 52% with stage IA, IB, and IIA, 76% with stage IIB, 85% with stage III, and 89% with stage IV. Fewer patients had elevated plasma YKL-40 (29%, 26%, 26%, and 46% for the different stages) and IL-6 (41%, 39%, 44%, and 66%) (Supplementary Table 1). Plasma YKL-40 and IL-6 were elevated in 35% and 42% of PC patients with normal serum CA 19.9. Fifty-two percent of patients with PC with normal CA 19.9 had either elevated plasma YKL-40 or IL-6. Ninety-two percent of all patients with PC had increased concentration of at least one of these three biomarkers.

**Figure 1:**
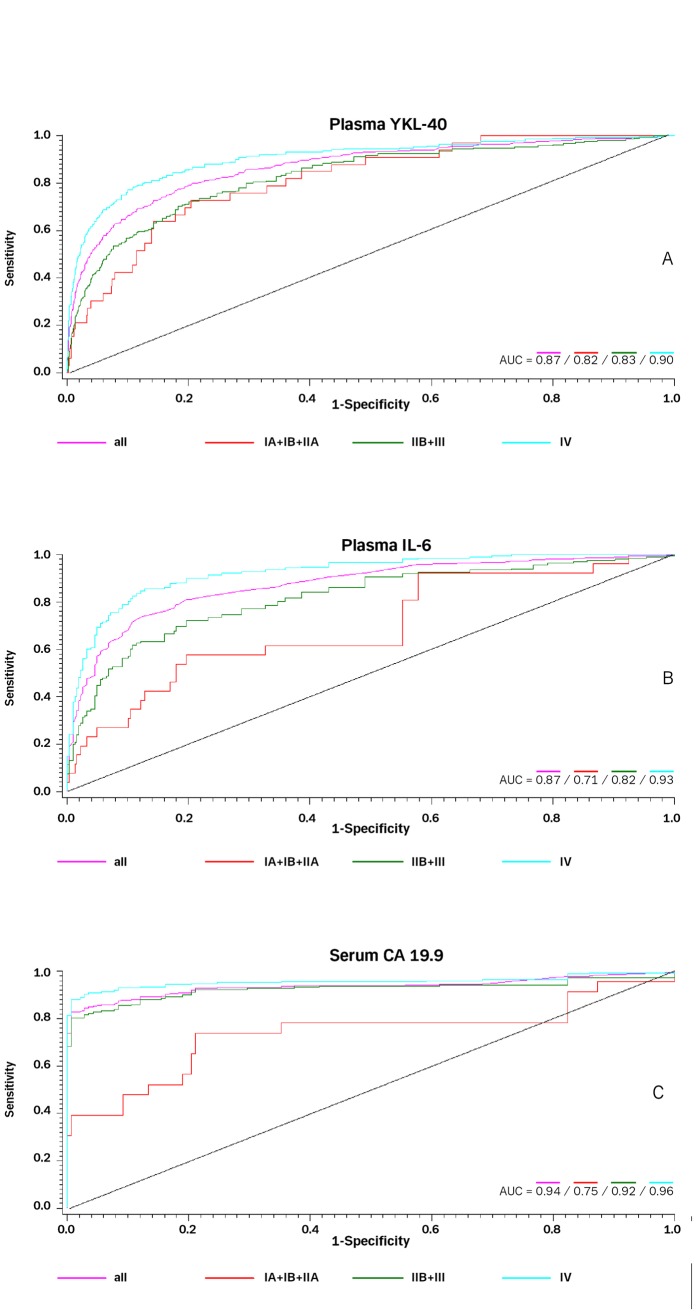
ROC curves for the diagnostic strength to identify PC using plasma YKL-40 (1A), plasma IL-6 (1B), or serum CA 19.9 (1C).

The OR for prediction of PC in the whole study population was significant for all three biomarkers, with serum CA 19.9 having the highest AUC (serum CA 19.9: OR = 2.28, 95% confidence interval (CI), 1.97 to2.68, p<0.0001, AUC = 0.94; plasma YKL-40: OR = 4.50, 3.99–5.08, p<0.0001, AUC = 0.87; and plasma IL-6: OR = 3.68, CI 3.08 to 4.44, p<0.0001, AUC = 0.87). The ROC curves are shown in Figure 1.

In patients undergoing surgery for PC, pre-treatment plasma YKL-40 correlated with plasma IL-6 (rho = 0.45, p<0.0001), serum CA 19.9 (rho = 0.40, p<0.0001) and age (rho = 0.40, p<0.0001). Lower correlations were found between plasma IL-6 and CA 19.9 (rho = 0.21, p = 0.03) and age (rho = 0.18, p = 0.052) and between serum CA 19.9 and age (rho = 0.29, p = 0.0021). In the patients not operated on, (stage IIB, III, and IV) pre-treatment plasma YKL-40 correlated with plasma IL-6 (rho = 0.51, p<0.0001) and age (rho = 0.21, p<0.0001), but not with serum CA 19.9 (rho = 0.079, p = 0.12). Low correlations were found between plasma IL-6 and CA 19.9 (rho = 0.22, p<0.0001). No correlations were found between age and plasma IL-6 (rho = 0.03) and serum CA 19.9 (rho = 0.02).

### YKL-40, IL-6 and CA 19.9 in Patients with Chronic Pancreatitis

Patients with chronic pancreatitis had higher plasma YKL-40 (median 102 µg/l, 35% had elevated level compared to upper normal level), IL-6 (3.5 ng/l, 27%) and serum CA 19-9 (15 KU/l, 25%) compared to healthy subjects. Patients with chronic pancreatitis had significantly lower concentrations of all three biomarkers compared to patients with PC (all stages combined) (YKL-40 p = 0.047; IL-6 p<0.0001; CA 19-9 p<0.0001) (Supplementary Figure 1).

### OS and Pre-treatment YKL-40, IL-6 and CA 19.9 in Patients Operated for PC

Univariate Cox analysis of patients undergoing surgery for PC showed that elevated pre-treatment plasma IL-6 and serum CA 19.9 (dichotomized according to normal levels), but not plasma YKL-40 were associated with short OS, Table 2. Multivariate Cox analysis including plasma YKL-40, IL-6, serum CA 19.9, stage, age, and gender showed that IL-6 and CA 19.9 (dichotomized according normal levels) were independent biomarkers of OS, Table 2. Similar results were found if IL-6 and CA 19.9 were included as continuous log-transformed values (Table 2).

**Table 2:** Univariate and multivariate Cox analyses of OS in 103 patients operated for PC according to pre-treatment concentrations of plasma YKL-40, IL-6, serum CA 19-9, age, sex, and stage.

	Univariate			Multivariate 1			Multivariate 2		
	HR	95% CI	P-value	HR	95% CI	P-value	HR	95% CI	P-value
Age pr 10 years	1.27	1.02–1.56	0.030	1.17	0.88–1.56	0.29	1.26	0.94–1.68	0.13
Sex F vs. M	0.68	0.45–1.04	0.074	0.79	0.46–1.36	0.40	0.74	0.43–1.29	0.29
Stage#	1.65	0.98–2.77	0.060	1.07	0.52–2.24	0.85	1.12	0.54–2.32	0.76
YKL-40Hi vs. loN = 142^§^	1.49	0.93–2.38	0.084	0.69	0.36–1.33	0.26			
IL-6Hi vs. loN = 122^§§^	2.40	1.50–3.86	0.0003	2.03	1.11–3.70	0.021			
Ca19.9Hi vs. loN = 107^§§§^	3.15	1.70–5.85	0.0003	2.51	1.22–5.15	0.013			
YKL-40Log2N = 142	1.20	1.01–1.42	0.041				0.89	0.65–1.21	0.44
IL6Log2N = 122	1.16	1.06–1.28	0.0021				1.15	1.00–1.32	0.056
Ca19-9Log2N = 107	1.14	1.05–1.23	0.0013				1.12	1.00–1.24	0.042

HR = Hazard ratio. CI = Confidence interval. Stage is scored as IA+IB+IIA vs. IIB+III. Hi, high concentration; Lo, low concentration.

Multivariate 1: Dichotomized biomarkers. ^§^Plasma YKL-40 was dichotomized (high vs. normal according to the age-corrected upper 95% percentage limit of YKL-40 in healthy subjects). ^§§^Plasma IL-6 was dichotomized (high vs. normal according to 95% percentage limit of IL-6 in healthy subjects, i.e. cut-off 4.5 ng/l). ^§§§^Serum CA 19.9 was dichotomized (high vs. normal according to the cut-off 37 KU/l).

Multivariate 2: The biomarkers are log transformed (log base 2) i.e. hazards are for a twofold difference.

**Figure 2:**
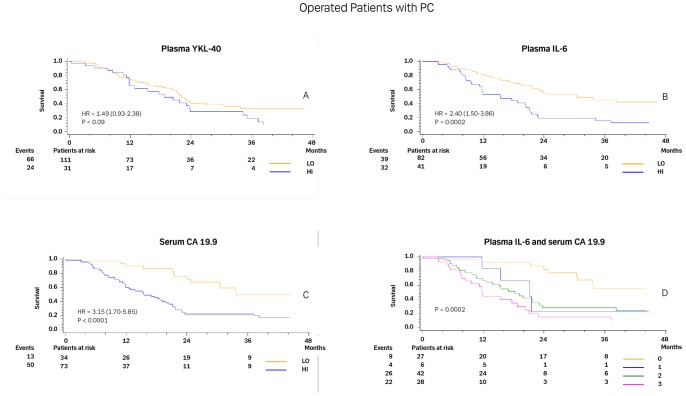
Kaplan-Meier survival curves showing the association between pre-treatment plasma YKL-40 (A), plasma IL-6 (B) and serum CA 19.9 (C) in patients operated on for PC. D shows the survival curves for no elevated biomarkers (yellow), only elevated IL-6 (blue), only elevated CA 19.9 (green), or both IL-6 and CA 19.9 elevated (purple). The P-value refers to the log-rank test for equality of strata. Patients were dichotomized by the upper normal level for each biomarker.

Figure 2 illustrates the Kaplan-Meier curves for patients undergoing surgery according to normal or elevated plasma YKL-40 (2A), IL-6 (2B), and serum CA 19.9 levels (2C). Patients with normal IL-6 and CA 19.9 had significantly longer OSs compared to patients with elevated levels. Figure 2D shows that the median OS was only 11.8 months (95% CI, 7.8 to18.8; and 45% alive after 1 year (95% CI, 25 to 63%)) if both IL-6 and CA 19.9 were elevated. The median survival was not reached in the group of patients with both normal IL-6 and CA 19.9 (first quartile 30.4 months, 95% CI, 11.0 to 33.7; with 92% alive after 1 year (95% CI, 71 to 98%)). The log-rank statistic demonstrated a significant difference (p<0.005) between survivals in these two groups.

#### OS and pre-treatment YKL-40, IL-6, and CA 19.9 in patients with PC not undergoing surgery

Univariate Cox analysis of patients with PC not undergoing surgery showed that elevated pre-treatment plasma YKL-40, IL-6, and serum CA 19.9 (dichotomized according to normal levels) were associated (p<0.0001) with short OS, Table 3. Multivariate Cox analysis including YKL-40, IL-6, CA 19.9, stage, age, and gender showed that YKL-40, IL-6, and CA 19.9 (dichotomized according normal levels) were independent biomarkers of OS, Table 3. Similar results were found if YKL-40, IL-6 and CA 19.9 were included as continuous log-transformed values (Table 3).

**Table 3:** Univariate and multivariate Cox analyses of OS in 370 patients with PC not undergoing surgery according to pre-treatment concentrations of plasma YKL-40, IL-6, serum CA 19.9, age, sex and stage.

	Univariate			Multivariate 1			Multivariate 2		
	HR	95% CI	P-value	HR	95% CI	P-value	HR	95% CI	P-value
Age pr 10 years	1.16	1.03–1.32	0.018	1.19	1.05–1.36	0.008	1.20	1.05–1.37	0.011
Sex F vs. M	0.89	0.71–1.11	0.28	0.92	0.72–1.16	0.47	0.94	0.74–1.18	0.58
Stage#	1.89	1.46–2.44	<0.0001	1.65	1.27–2.15	<0.0001	1.35	1.02–1.77	0.034
YKL-40Hi vs. loN = 142^§^	1.60	1.28–2.00	<0.0001	1.30	1.03–1.64	0.029			
IL-6Hi vs. loN = 122^§§^	2.16	1.70–2.74	<0.0001	1.71	1.33–2.20	<0.0001			
Ca19.9Hi vs. loN = 107^§§§^	1.72	1.20–2.48	0.0035	1.54	1.06–2.24	0.022			
YKL-40Log2N = 142	1.32	1.22–1.44	<0.0001				1.13	1.02–1.24	0.015
IL6Log2N = 122	1.35	1.25–1.45	<0.0001				1.23	1.13–1.35	<0.0001
Ca19-9Log2N = 107	1.10	1.07–1.14	<0.0001				1.09	1.05–1.12	<0.0001

HR = Hazard ratio. CI = Confidence interval. Stage is scored as IIB+III vs. IV. Hi, high concentration; Lo, low concentration.

Multivariate 1: Dichotomized biomarkers. ^§^Plasma YKL-40 was dichotomized (high vs. normal according to the age-corrected upper 95% percentage limit of YKL-40 in healthy subjects). ^§§^Plasma IL-6 was dichotomized (high vs. normal according to 95% percentage limit of IL-6 in healthy subjects, i.e. cut-off 4.5 ng/l). ^§§§^Serum CA 19.9 was dichotomized (high vs. normal according to the cut-off 37 kU/l).

Multivariate 2: The biomarkers are log transformed (log base 2) i.e. hazards are for a twofold difference.

**Figure 3:**
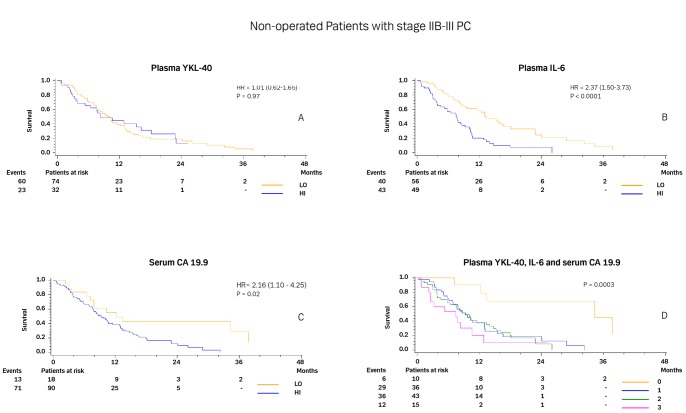
Kaplan-Meier survival curves showing the association between pre-treatment plasma YKL-40 (A), plasma IL-6 (B), and serum CA 19.9 (C) in patients with PC stage IIB and III. D shows the survival curves for 0–3 elevated biomarkers. The P-value refers to the log-rank test for equality of strata. Patients were dichotomized by the upper normal level for each biomarker.

Figure 3 illustrates the Kaplan-Meier curves for patients with stage IIB and III not undergoing surgery according to normal or elevated plasma YKL-40 (3A), IL-6 (3B) and serum CA 19.9 levels (3C). Patients with normal YKL-40, IL-6, and CA 19.9 had significantly longer OS than did patients with elevated levels. Figure 3D illustrates that the median OS was only 7.5 months (95% CI, 2.3 to 10.6; and 20% alive after 1 year) if all three biomarkers were elevated versus 34.4 months (7.2 to 51.1; and 90% alive after 1 year) if all three biomarkers were normal (p = 0.003).

**Figure 4:**
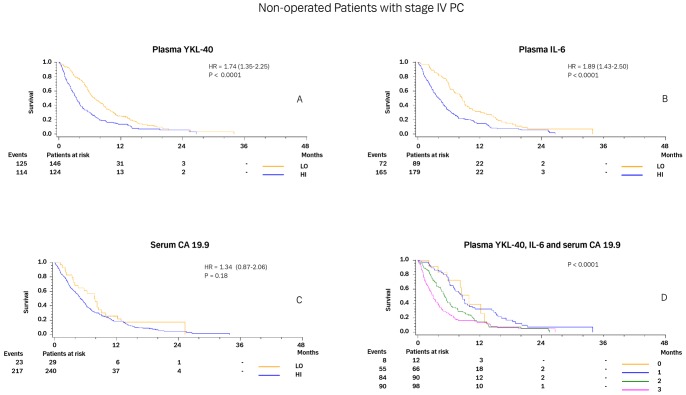
Kaplan-Meier survival curves showing the association between pre-treatment plasma YKL-40 (A), plasma IL-6 (B) and serum CA 19.9 (C) in non-operated patients with PC stage IV. D shows the survival curves for 0–3 elevated biomarkers. The P-value refers to the log-rank test for equality of strata. Patients were dichotomized by the upper normal level for each biomarker.

Figure 4 illustrates the Kaplan-Meier curves for patients with stage IV not undergoing surgery according to normal or elevated plasma YKL-40 (4A), IL-6 (4B), and serum CA 19.9 levels (4C). Patients with normal YKL-40, IL-6, and CA 19.9 had significantly longer OS than patients with elevated levels. Figure 4D illustrates that the median OS was only 3.0 months (2.2–3.9; and 13% alive after 1 year) if all three biomarkers were elevated versus 10.0 months (3.9–12.9; and 39% alive after 1 year) if all three biomarkers were normal (p<0.0001).

Supplementary Figure 2 shows the Kaplan-Meier curves for all patients with stages IIB, III, and IV combined.

## Discussion

We investigated the diagnostic and prognostic value of plasma concentrations of YKL-40 and IL-6 in 559 patients with PC included in two large prospective biomarker studies of patients with PC from Denmark and Heidelberg. The results were compared with those of serum CA 19.9. Thirty-six percent had elevated YKL-40, and 54% of all patients with PC had elevated plasma IL-6. Although the levels of both biomarkers increased with increasing tumor stage, they cannot be used alone for early diagnosis of PC, and serum CA19.9 had highest OR for prediction of PC.

IL-6 stimulates production of YKL-40, and a correlation was found between these two biomarkers. Plasma YKL-40 and IL-6 could be valuable diagnostic biomarkers in Lewis antigen-negative patients with PC and normal serum concentrations of CA 19.9 [29], since 52% of these patients had elevated YKL-40 or IL-6. The tumor volume in PC is often modest, but the frequency of patients with elevated plasma YKL-40 is higher than in patients with other solid tumors and hematologic malignancies [5], [34]. A higher percentage of patients with elevated plasma YKL-40 [35] is seen only in ovarian cancer.

High plasma concentrations of YKL-40 and IL-6 are independent prognostic biomarkers associated with short OS in patients with many different types of cancer, but only a few studies have evaluated the prognostic value of plasma YKL-40 and IL-6 in patients with PCs [34]. The main results of our study of patients with PC are that high (i.e. compared to normal values) pre-operative plasma IL-6 and serum CA 19.9 levels were independent prognostic biomarkers of short OS. If both plasma IL-6 and serum CA 19.9 were elevated before surgery, the patients had shorter OS than patients with normal biomarkers level (45% vs. 92% alive after 1 year). Multivariate Cox analysis showed that plasma YKL-40, IL-6 and serum CA 19.9 were all independent prognostic biomarkers of OS in patients with locally advanced or metastatic PC. In these patients, high levels of all three biomarkers identified a subgroup of patients with a very short median survival, i.e. only 7.5 months, vs. 34.4 months if all biomarkers were normal in patients with stage IIB and III, and 3.0 months vs. 10.0 months if all biomarkers were normal in patients with stage IV. IL-6 stimulates YKL-40 production, and in most types of cancer the proportion of patients with elevated plasma concentrations of YKL-40 is much higher in patients with metastatic disease than in patients with localized disease [5], [10]. This may explain why plasma YKL-40 in the present study was an independent prognostic biomarker of OS in patients with locally advanced or metastatic PC but not in patients with localized PC that have been operated.

There is an increasing interest for the tumorigenic microenvironment [36], [39]. An important part of this microenvironment is a tumor promoting inflammation and a varying density of infiltration of immune cells [36]. PC is characterized by scattered cancer cells embedded in a fibrotic desmoplastic stroma [40]. Neither YKL-40 nor IL-6 is a cancer-specific biomarker, and both are produced by cancer cells and inflammatory cells and are biomarkers of inflammation and tissue remodeling [3], [5], [6], [14], [15], [41]–[43]. Elevated plasma YKL-40 is found in a subset of patients with cancer and patients with non-malignant diseases characterized by inflammation and/or tissue remodeling [5]. Recent studies have implicated YKL-40 in several biological processes such as inflammation, angiogenesis, apoptosis, cell proliferation, differentiation, and regulation of extracellular tissue remodeling [5], [7]–[21], all processes important for the progression of cancer cell growth, tumor angiogenesis, and metastatic potential [36]–[38]. However, further studies are required to completely understand the functions of YKL-40 in cancer development and progression. In the general population, elevated plasma YKL-40 predicts increased risk of gastrointestinal cancer and a poor OS in these patients [23], but serum YKL-40 has little diagnostic value for early diagnosis of hepato-biliary cancer [44].

IL-6 is produced by cancer cells, including PC, macrophages, lymphocytes, and endothelial cells [6], [24]–[26]. IL-6 plays a role in inflammation and the acute-phase response, acts as a paracrine and autocrine growth factor for cancer cells, and inhibits radio- and chemotherapy-induced apoptosis of PC cells [6], [25], [27], [28], [43]. The presence of stromal desmoplasia is a hallmark in PC, driven by the pancreatic stellate cells and its interactions with cytokines like IL-6 [45]. Cachexia in patients with PC and other cancers is also regulated by IL-6 [24], [46], [47].

Recently, genome-wide association studies [48], [49] have identified SNPs (single nucleotide ploymophisms) associated with susceptibility to PC. Some SNPs are related to plasma concentrations of CA 19.9 in healthy subjects, but no association was found between these SNPs and PC. It is not known whether certain SNPs in the YKL-40 and IL-6 genes are associated with PC.

The strength of our study is its prospective design and that YKL-40 and IL-6 measurements in plasma were determined blindly, i.e. without knowledge of clinical data and the OS of the patients. Misclassification of YKL-40 and IL-6 levels will always occur to some extent even though we measured all samples in duplicate and had low coefficients of variation. The pre-treatment plasma concentrations of YKL-40 in the patients with PC were dichotomized according to the 95% upper normal YKL-40 level in a large group of 3,130 healthy subjects from the Danish general population [31]. Since plasma YKL-40 increases with age, we used age-corrected plasma YKL-40 levels. The cohort of our normal subjects is quite unique; they had no known disease at time of blood sampling in 1991–1994 and remained healthy and alive during the 16-year follow-up period [30]. We therefore consider that our classifications of PC patients with normal and elevated plasma YKL-40 are correct. Plasma concentrations of IL-6 do not increase with age, and we think that the number of healthy subjects used to determine the normal range of plasma IL-6 is sufficient.

In conclusion, plasma YKL-40 and IL-6 alone are not useful as new diagnostic biomarkers to identify patients with PC at an early stage or to discriminate between patients with PC and chronic pancreatitis. Serum CA 19-9 was the best diagnostic biomarker. However, plasma concentrations of YKL-40 and IL-6 may provide information in the subgroup of patients with PC and normal serum CA 19.9, since YKL-40 or IL-6 was elevated in 52% of these patients. In the group of patients with PC undergoing to surgery, a high pre-operative plasma IL-6 and serum CA 19.9 identified a sub-group of patients with very short survival. Future studies could test whether these patients may benefit from neo-adjuvant chemotherapy. Ninety-two percent of our patients with locally advanced or metastatic PC were treated with first line palliative monotherapy with gemcitabine, and if all three biomarkers were elevated in these patients, their prognosis was dismal. It is not known whether this subgroup of patients with PC and very poor prognosis could have benefited from a more aggressive therapy, such as FOLFIRINOX or abraxane.

## Supporting Information

Figure S1 **Box-plots of pre-treatment plasma YKL-40 (A), plasma IL-6 (B) and serum CA 19.9 (C) in patients with PC according to stage and in patients with chronic pancreatitis.** The median value is the line in the middle of the box and the 25^th^ and 75^th^ percentile are the lower and upper part of the box. The whiskers are the 5^th^ and 95^th^ percentiles. Outliers are given as dots.
(PDF)

Figure S2 **Kaplan-Meier survival curves showing the association between pre-treatment plasma YKL-40 (A), plasma IL-6 (B) and serum CA 19.9 (C) in non-operated patients with PC stage IIB, III and IV.** D shows Kaplan-Meier survival curves for 0–3 elevated biomarkers. The P-value refers to the log-rank test for equality of strata. Patients were dichotomized by the upper normal level for each biomarker.
(PDF)

Table S1 **Pre-treatment plasma YKL-40, IL-6 and serum CA 19.9 in patients with PC according to stage.**
(DOCX)
