# Supplementary figures and images for: Diagnostic and Prognostic Impact of Circulating YKL-40, IL-6, and CA 19.9 in Patients with Pancreatic Cancer
Source: PLoS One. 2013 Jun 26;8(6):e67059. doi: 10.1371/journal.pone.0067059 (PMC3694124; doi:10.1371/journal.pone.0067059)

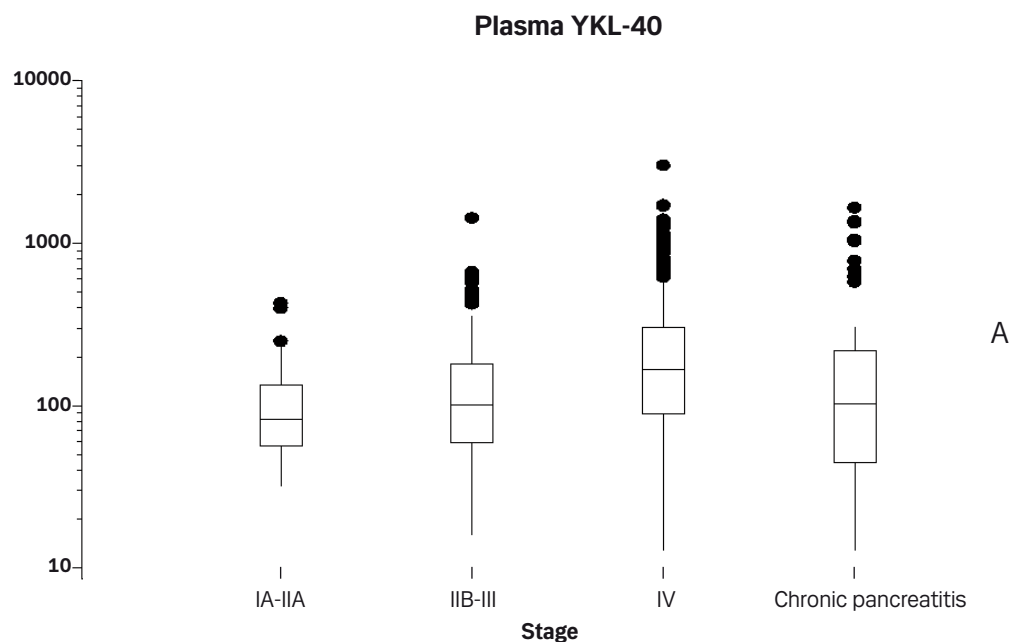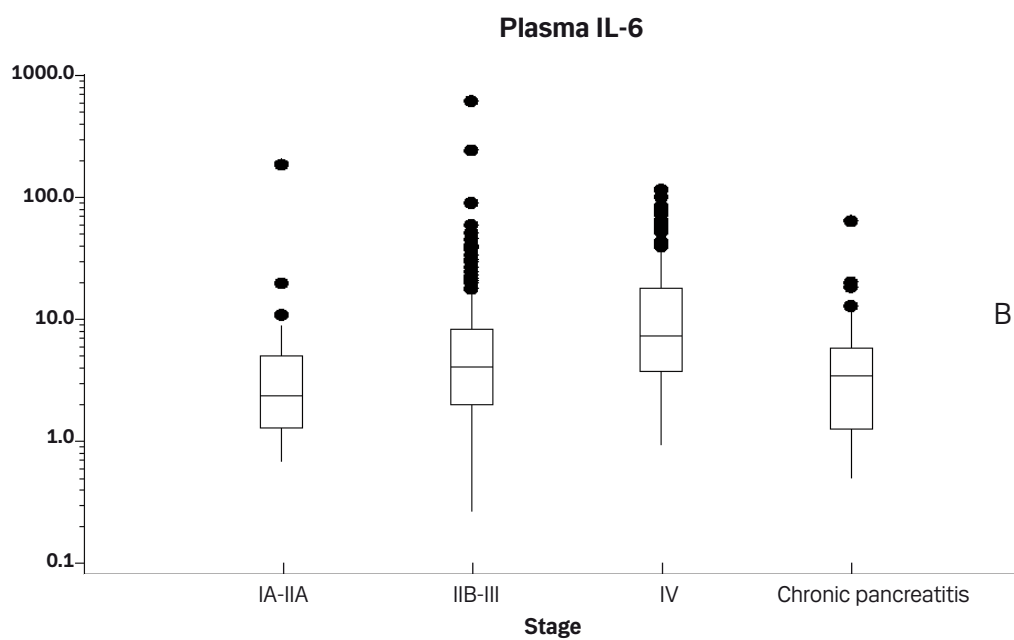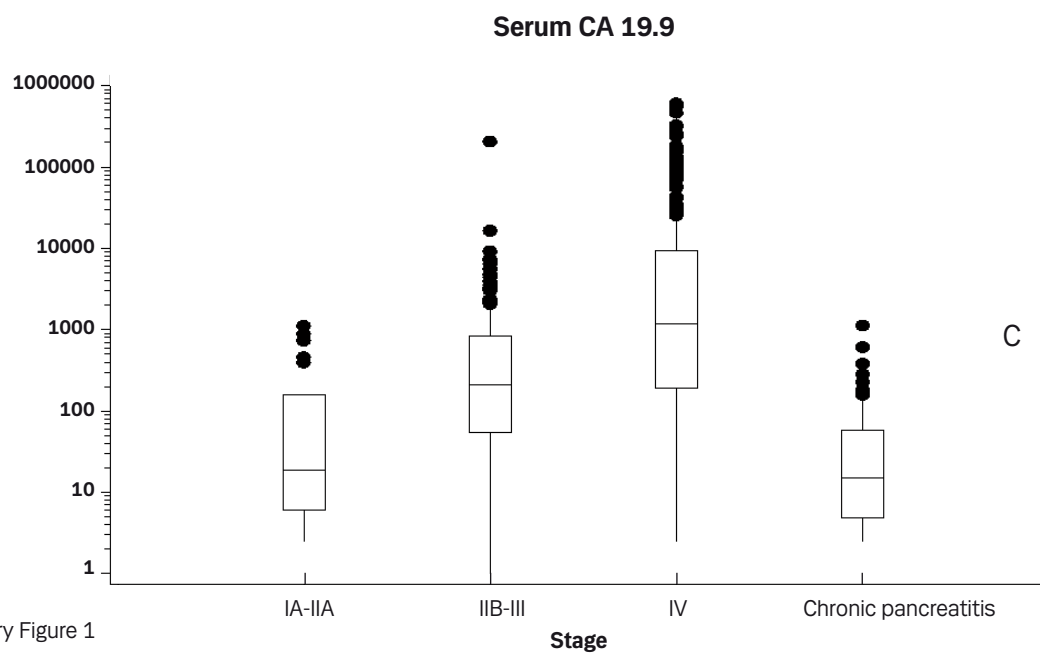

Supplement: Figure S1 — Box-plots of pre-treatment plasma YKL-40 (A), plasma IL-6 (B) and serum CA 19.9 (C) in patients with PC according to stage and in patients with chronic pancreatitis. The median value is the line in the middle of the box and the 25th and 75th percentile are the lower and upper part of the box. The whiskers are the 5th and 95th percentiles. Outliers are given as dots. (PDF) [file pone.0067059.s001.pdf]

## Non-operated Patients with PC

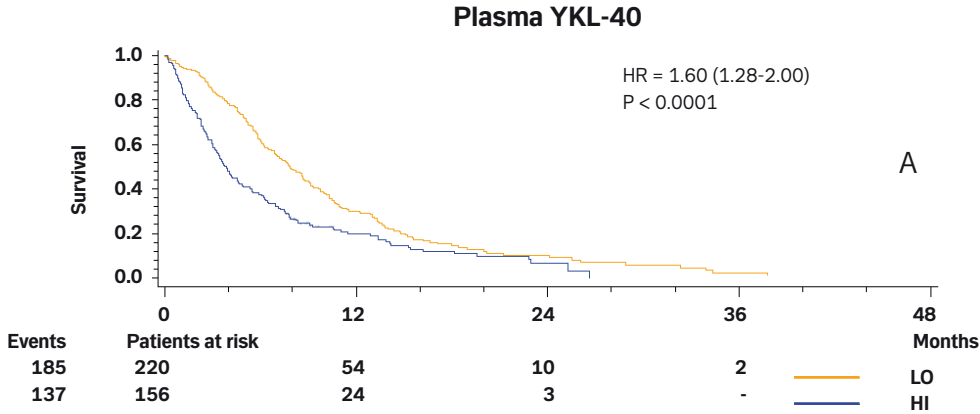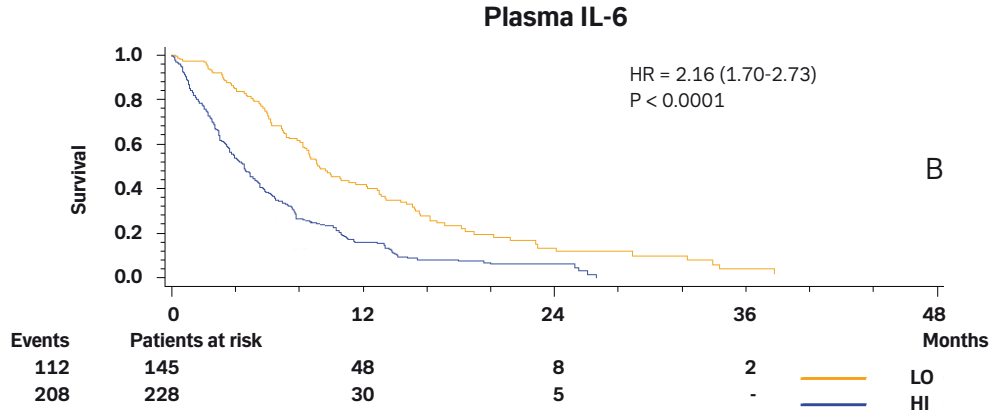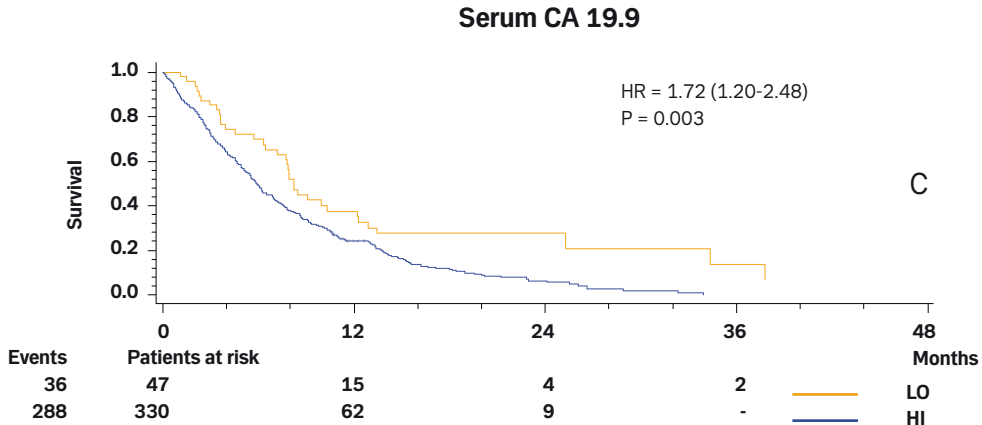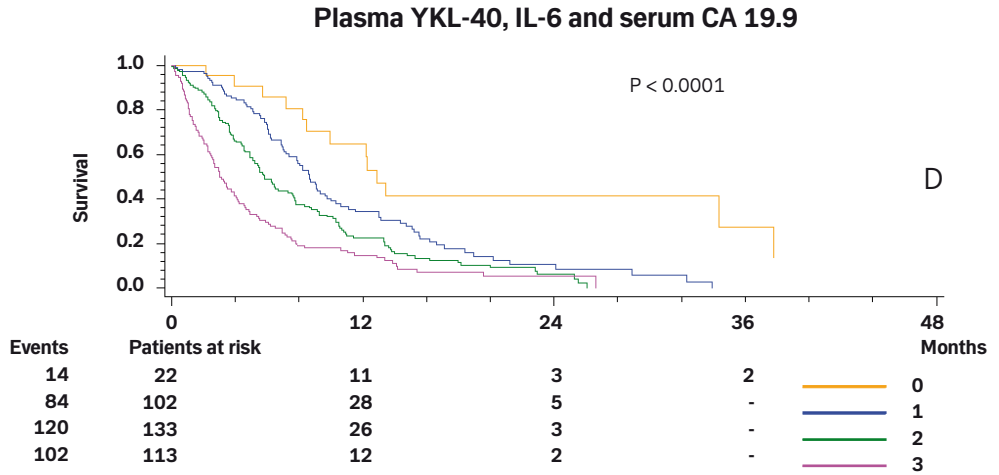

Supplementary Figure 2

Supplement: Figure S2 — Kaplan-Meier survival curves showing the association between pre-treatment plasma YKL-40 (A), plasma IL-6 (B) and serum CA 19.9 (C) in non-operated patients with PC stage IIB, III and IV. D shows Kaplan-Meier survival curves for 0–3 elevated biomarkers. The P-value refers to the log-rank test for equality of strata. Patients were dichotomized by the upper normal level for each biomarker. (PDF) [file pone.0067059.s002.pdf]
