# Supplementary material for: Diagnostic and Prognostic Impact of Circulating YKL-40, IL-6, and CA 19.9 in Patients with Pancreatic Cancer
Source: PLoS One. 2013 Jun 26;8(6):e67059. doi: 10.1371/journal.pone.0067059 (PMC3694124; doi:10.1371/journal.pone.0067059)
Supplement: Table S1 — Pre-treatment plasma YKL-40, IL-6 and serum CA 19.9 in patients with PC according to stage. (DOCX) [file pone.0067059.s003.docx]

**Supplementary Table 1.** Pre-treatment plasma YKL-40, IL-6 and serum CA 19.9 in patients with PC according to stage.

|  | **Number** | **Median (IQR)** | **Percentage of patients with elevated biomarker** |
| --- | --- | --- | --- |
| **YKL-40 Stage IA, IB, IIA** | 55 | 100 μg/l (65 - 192) | 29% |
| **YKL-40 Stage IIB** | 125 | 99 μg/l (59 - 181) | 26 % |
| **YKL-40 Stage III** | 90 | 104 μg/l (63 - 174) | 26% |
| **YKL-40 Stage IV** | 273 | 166 μg/l (89 - 301) | 46% |
| **IL-6 Stage IA, IB, IIA** | 49 | 3.3 ng/l (1.9 - 8.8) | 41% |
| **IL-6 Stage IIB** | 113 | 3.4 ng/l (1.8 - 9.0) | 39% |
| **IL-6 Stage III** | 88 | 4.7 ng/l (2.4 - 7.7) | 44% |
| **IL-6 Stage IV** | 271 | 7.4 ng/l (3.6 - 18) | 66% |
| **CA 19-9 Stage IA, IB, IIA** | 46 | 53 KU/l (10 - 622) | 52% |
| **CA 19-9 Stage IIB** | 101 | 144 KU/l (43 - 702) | 76% |
| **CA 19-9 Stage III** | 91 | 328 KU/l (65 - 990) | 85% |
| **CA 19-9 Stage IV** | 270 | 1050 KU/l (150 - 9178) | 89% |

¤ The 95^th^ percentile of plasma YKL-40 and IL-6 levels in healthy subjects (age-corrected) was used as cut-off (age-corrected). The cut-off for serum CA 19-9 was 37 KU/l. IQR = interquartile range.
